# Antitumoral effects of Bortezomib in malignant mesothelioma: evidence of mild endoplasmic reticulum stress in vitro and activation of T cell response in vivo

**DOI:** 10.1186/s13062-023-00374-w

**Published:** 2023-04-17

**Authors:** Monica Benvenuto, Valentina Angiolini, Chiara Focaccetti, Daniela Nardozi, Camilla Palumbo, Raffaele Carrano, Alessandra Rufini, Riccardo Bei, Martino Tony Miele, Patrizia Mancini, Giovanni Barillari, Mara Cirone, Elisabetta Ferretti, Grazia Raffaella Tundo, Luciano Mutti, Laura Masuelli, Roberto Bei

**Affiliations:** 1 Department of Clinical Sciences and Translational Medicine, University of Rome “Tor Vergata”, Rome, Italy; 2 Saint Camillus International, University of Health and Medical Sciences, Rome, Italy; 3 Department of Experimental Medicine, Sapienza University of Rome, Rome, Italy; 4 Department of Biomedicine and Prevention, University of Rome “Tor Vergata”, Rome, Italy; 5 Medical School, University of Rome “Tor Vergata”, Rome, Italy; 6 Department of Experimental Medicine, University of Rome “Tor Vergata”, Rome, Italy; 7 Department of Biotechnological and Applied Clinical Sciences, University of L’Aquila, L’Aquila, Italy; 8 Center for Biotechnology, Sbarro Institute for Cancer Research and Molecular Medicine, College of Science and Technology, Temple University, Philadelphia, PA, USA

**Keywords:** Malignant mesothelioma, Bortezomib, ER stress, Mice, Proteasome

## Abstract

**Background:**

Malignant mesothelioma (MM) is a rare tumor with a dismal prognosis. The low efficacy of current treatment options highlights the urge to identify more effective therapies aimed at improving MM patients’ survival. Bortezomib (Bor) is a specific and reversible inhibitor of the chymotrypsin-like activity of the 20S core of the proteasome, currently approved for the treatment of multiple myeloma and mantle cell lymphoma. On the other hand, Bor appears to have limited clinical effects on solid tumors, because of its low penetration and accumulation into tumor tissues following intravenous administration. These limitations could be overcome in MM through intracavitary delivery, with the advantage of increasing local drug concentration and decreasing systemic toxicity.

**Methods:**

In this study, we investigated the effects of Bor on cell survival, cell cycle distribution and modulation of apoptotic and pro-survival pathways in human MM cell lines of different histotypes cultured in vitro. Further, using a mouse MM cell line that reproducibly forms ascites when intraperitoneally injected in syngeneic C57BL/6 mice, we investigated the effects of intraperitoneal Bor administration in vivo on both tumor growth and the modulation of the tumor immune microenvironment.

**Results:**

We demonstrate that Bor inhibited MM cell growth and induced apoptosis. Further, Bor activated the Unfolded Protein Response, which however appeared to participate in lowering cells’ sensitivity to the drug’s cytotoxic effects. Bor also affected the expression of EGFR and ErbB2 and the activation of downstream pro-survival signaling effectors, including ERK1/2 and AKT. In vivo, Bor was able to suppress MM growth and extend mice survival. The Bor-mediated delay of tumor progression was sustained by increased activation of T lymphocytes recruited to the tumor microenvironment.

**Conclusions:**

The results presented herein support the use of Bor in MM and advocate future studies aimed at defining the therapeutic potential of Bor and Bor-based combination regimens for this treatment-resistant, aggressive tumor.

**Supplementary Information:**

The online version contains supplementary material available at 10.1186/s13062-023-00374-w.

## Background

Malignant mesothelioma (MM) is a rare tumor arising from the mesothelial cells that line the serosal cavities, the most common site of origin being the pleura, followed by the peritoneal membrane (~ 80–85% and 10–15% of cases, respectively) [1, 2]. While the main risk factor for MM is asbestos exposure, a relationship between MM development and chronic serous inflammation or exposure to different mineral fibers or to ionizing radiations has also been reported; further, genetic predisposing factors for MM have recently been identified [1, 3, 4].

With a mean overall survival from diagnosis of about 1 year and a 5-year survival rate of about 5–10% for all tumor stages, the prognosis for MM patients is presently poor [1, 5]. Besides the aggressive and chemoresistant nature of MM, other factors which contribute to its dismal prognosis are the delayed onset of symptoms and late diagnosis as well as the anatomical location of the tumor, which limits both its macroscopic clearance and radical radiotherapy interventions [1, 2, 5–7]. As for chemotherapy, with the currently approved combination of platinum agents and folate antimetabolites, such as pemetrexed, the median survival of MM patients ranges between 4 and 19 months, depending on tumor histology [1, 5]. A small benefit in terms of increased survival can be obtained with the addition of the Vascular Endothelial Growth Factor inhibitor Bevacizumab to the previous regimen, allowing a median survival of 19 months vs. the 16 months achieved with platinum agents/folates alone [1]. MM is further characterized by an immunosuppressive microenvironment, and modest response rates have been obtained with immunotherapy approaches, including those based on the recently introduced Immune Checkpoint Inhibitors (ICIs) [1, 2, 5, 8, 9]. The low efficacy of current treatment options highlights the urge to identify more effective therapies aimed at improving MM patients’ survival.

The proteasome is a multi-catalytic protease complex that degrades ubiquitin-tagged proteins and plays a main role both in the removal of damaged/misfolded proteins and in the regulated turnover of short-lived signaling molecules involved in multiple processes, among which are cell cycle progression, cell survival and apoptosis [10–12]. While proteasome inhibition generally leads to cell cycle arrest, endoplasmic reticulum (ER) stress and apoptotic cell death, the observation that cancer cells have an increased dependence on proteasome activity and are more sensitive than non-transformed cells to the effects of proteasome inhibitors has provided the rational basis for using these agents in antineoplastic therapy [13, 14]. That cancer cells have an increased demand for proteasomal protein degradation is for instance supported by the finding that constitutive proteasome activity is upregulated in different cancer types [15]. In addition, a sustained protein turnover appears to be required to meet the degradative needs of rapidly proliferating cancer cells [13]. Moreover, many proteins critically involved in cancer cells survival and proliferation are known targets of proteasomal degradation, as it has been demonstrated for cyclins, the cyclin-dependent kinase inhibitor p27, the p53 tumor suppressor, the IκB-α inhibitor of NF-κB and the pro-apoptotic BH3-only and Bax proteins [11, 14].

Bortezomib (Bor), a dipeptide boronic acid that acts as a specific and reversible inhibitor of the chymotrypsin-like activity of the 20S core of the proteasome, is the first proteasome inhibitor that entered clinical trials and is currently approved for the treatment of multiple myeloma and mantle cell lymphoma [10, 11]. However, despite its efficacy on some hematological malignancies and despite promising preclinical data, Bor appears to have limited clinical effects on solid tumors, a finding which is ascribed to several factors, including its low penetration and accumulation into tumor tissues following intravenous (i.v.) administration [10, 14, 16]. In this regard, the regional features of MM, which has a highly locally invasive behavior but is characterized by a low metastatic efficiency [2, 17], make this tumor a good candidate for Bor and Bor-based combination therapies. Indeed, under the currently short survival expectancy of MM patients, morbidity and mortality are essentially caused by the extensive local spread of the tumor in the pleural/peritoneal cavity and the destructive infiltration of surrounding tissues, while distant metastasis, although more frequent than it was previously thought, do not appear to have evident prognostic implications [17]. Therefore, in MM the limitations associated with the low tissue distribution of Bor could be overcome through intracavitary delivery, with the advantage of increasing local drug concentration and decreasing systemic toxicity [18, 19].

Given these premises, we investigated the effects of Bor on cell survival, cell cycle distribution, and modulation of apoptotic and pro-survival pathways in human MM cell lines of different histotypes cultured in vitro. Further, using a mouse MM cell line that reproducibly forms ascites when intraperitoneally injected in syngeneic C57BL/6 mice, we investigated the effects of intracavitary Bor administration in vivo on both tumor growth and the modulation of the tumor immune microenvironment. The results presented herein support the use of Bor in MM and advocate future studies aimed at defining the therapeutic potential of Bor and Bor-based combination regimens for this treatment-resistant, aggressive tumor.

## Results

### Effects of Bortezomib on growth and death of MM cells

**Fig. 1:**
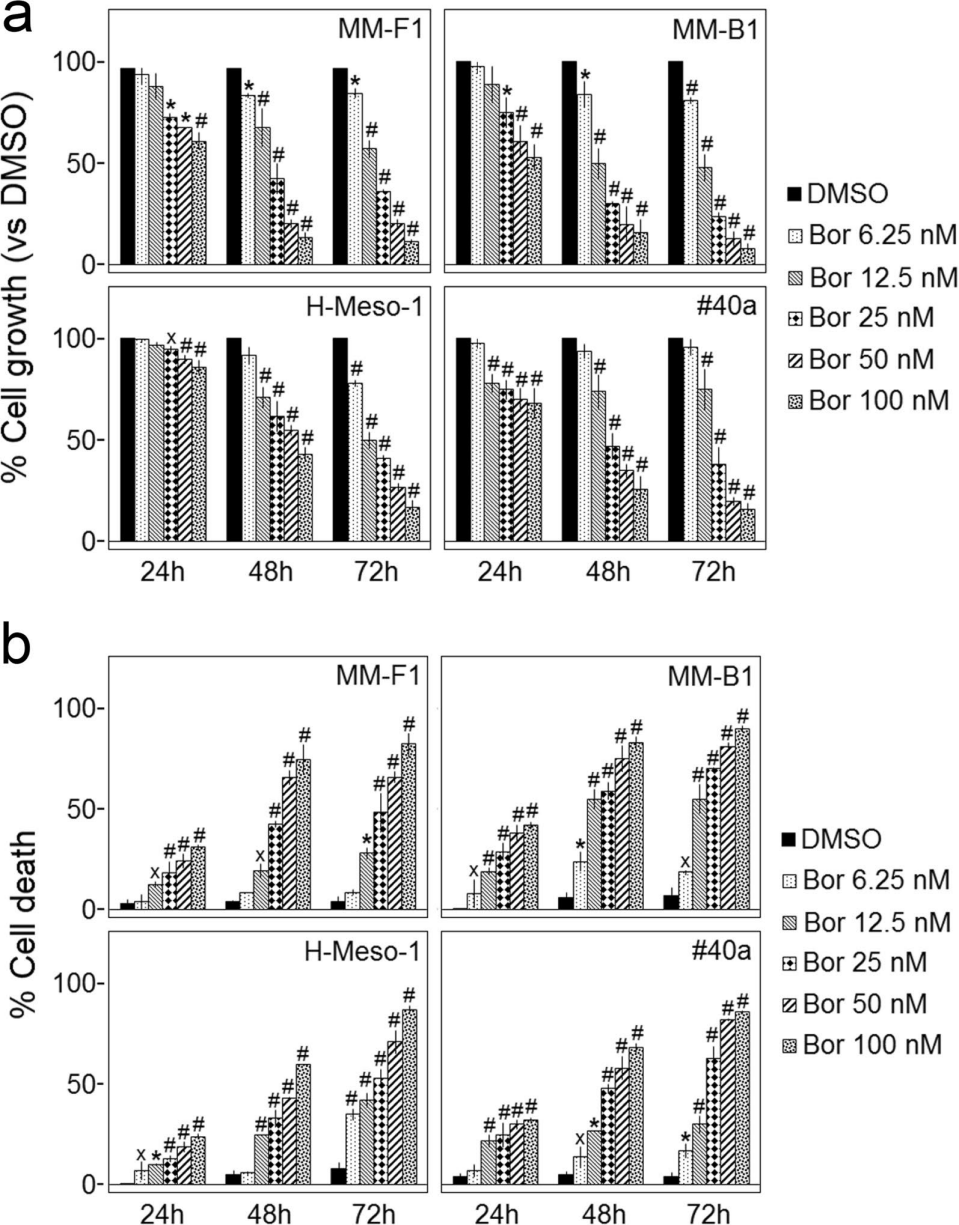
Effect of Bor on growth and death of MM cells. **a** The growth of human (MM-F1, MM-B1, H-Meso-1) and mouse (#40a) cell lines was assessed by the SRB assay after 24, 48 and 72 h of treatment with Bor or DMSO. The percentage of cell growth of Bor-treated cultures was calculated by normalizing their O.D. value to that of DMSO control cultures. The results are expressed as mean ± standard deviation (SD) of three independent experiments performed in triplicate (˟*p* ≤ 0.05; *p ≤ 0.01; #*p* ≤ 0.001 vs. DMSO). **b** Trypan blue exclusion assay was performed to determine the cell death percentage of human and mouse MM cells treated with Bor or DMSO for 24, 48, and 72 h. The results are expressed as the mean ± SD values of three independent experiments performed in triplicate (˟*p* ≤ 0.05; *p ≤ 0.01, #*p* ≤ 0.001 vs. DMSO)

Growth and death of human (H-Meso-1, MM-F1, MM-B1) and mouse (#40a) MM cells were evaluated by the Sulforhodamine B (SRB) and trypan blue exclusion assays after treatment with increasing concentrations of Bor (range 6.25–100 nM), or DMSO as vehicle control, for 24, 48 and 72 h. As assessed by SRB assay, Bor inhibited cell growth in a dose- and time-dependent manner in all cell lines. In particular, after 48 h of treatment the effect of Bor gained statistical significance at all the tested concentrations in MM-F1 and MM-B1 cells and at concentrations ≥ 12.5 nM in H-Meso-1 and #40a cells (Fig. 1a). The concentration of Bor that inhibits cellular growth by 50% (IC_50_) was also determined for each cell line (Table 1). After 72 h of treatment, MM-B1 was the most sensitive and H-Meso-1 was the less sensitive cell line.

**Table 1:** Bor concentrations at 50% of cell growth inhibition (IC_50_) on human and mouse MM cell lines

Cell line	Hours of Bor treatment	IC_50_ ± SD (nM)
MM-F1	48	21.7 ± 2.7
	72	18.2 ± 1.2
MM-B1	48	15.0 ± 1.0
	72	13.3 ± 0.8
H-Meso-1	48	62.3 ± 13.9
	72	16.9 ± 1.5
#40a	48	30.0 ± 3.9
	72	21.9 ± 3.8

As assessed by the trypan blue exclusion assay, Bor also increased the percentage of dead cells in a dose- and time-dependent manner in all MM cell lines, as compared to DMSO controls (Fig. 1b).

### Effects of Bortezomib on MM cells apoptosis and cell cycle distribution

**Fig. 2:**
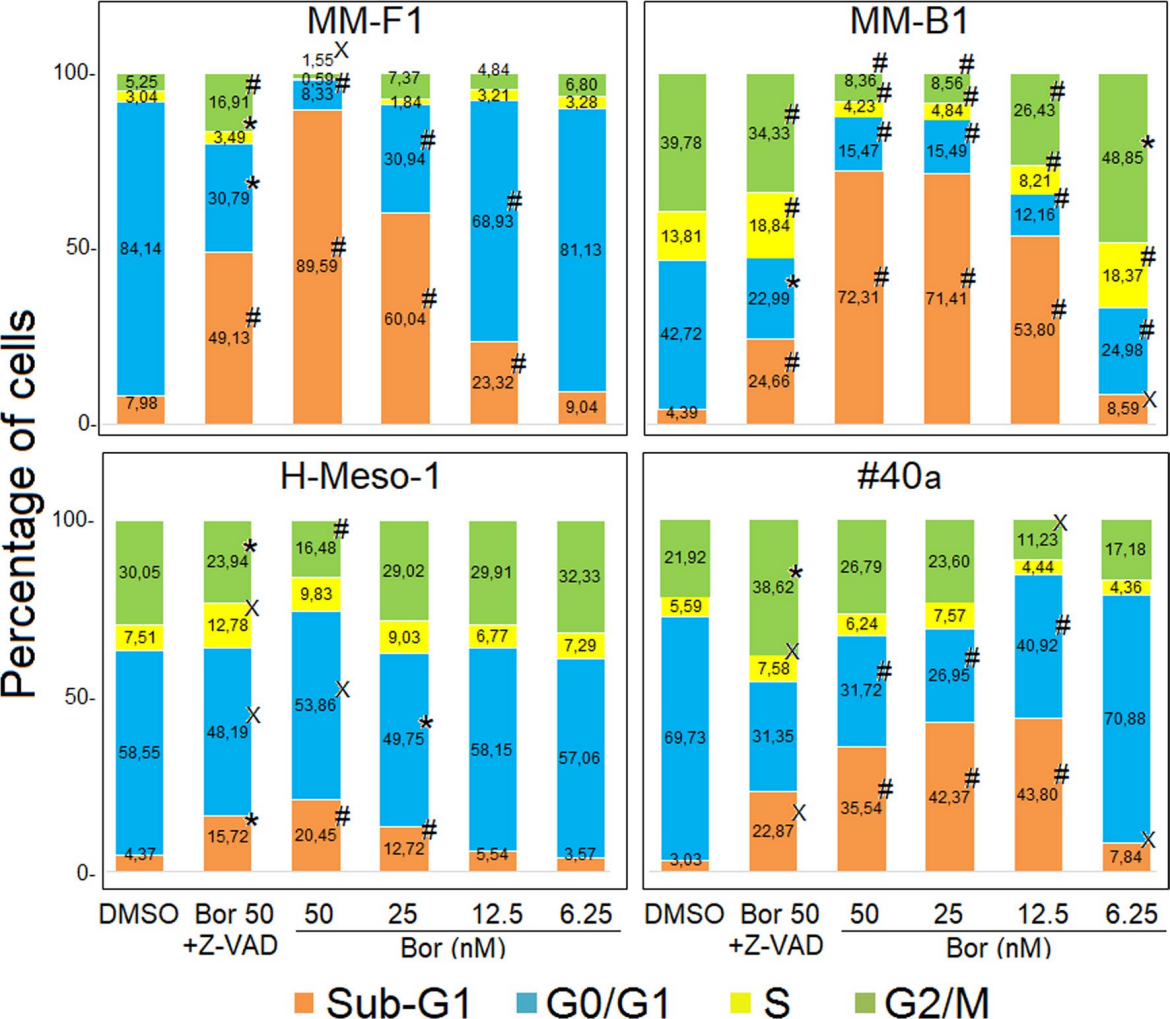
Effect of Bor on MM cells apoptosis and cell cycle distribution. Cells were treated with Bor at the indicated concentrations for 48 h and then analyzed by FACS analysis. Reported is the mean percentage of cells in sub-G1, G0/G1, S and G2/M phases obtained from two independent experiments. Significance of the effect of Bor vs. that of DMSO and significance of the effect of 50 nM Bor + Z-VAD-FMK vs. that of 50 nM Bor was calculated with one-way ANOVA (^x^*p* ≤ 0.05; *p ≤ 0.01, ^#^*p* ≤ 0.001)

In order to evaluate Bor effects on apoptosis and cell cycle distribution, flow cytometric analysis of the cellular DNA content was performed on MM cells treated with Bor or DMSO for 48 h. Bor increased the percentage of cells in the sub-G1 phase in all cell lines (Fig. 2). In particular, the percentage of cells in the sub-G1 phase increased with Bor ≥ 6.25 nM in MM-B1 and #40a cells, whereas in MM-F1 and H-Meso-1 a significant increase of the sub-G1 cell fraction was observed with Bor concentrations ≥ 12.5 and ≥ 25 nM, respectively (Fig. 2). On the whole, the increase in sub-G1 cells was accompanied by a decrease of the G0/G1 cell fraction in all cell lines, whereas S and G2/M cell fractions decreased in a cell-dependent manner and only at selected Bor concentrations.

Cells in the sub-G1 phase are characterized by a hypodiploid DNA content, which is typical, albeit not exclusive, of apoptosis. To confirm the effect of Bor in inducing MM cell apoptosis, cells were treated with Bor at the highest dose in the presence of the universal caspase inhibitor Z-VAD-FMK. In all cell lines, the percentage of cells in the sub-G1 phase was significantly reduced when Bor was used in combination with Z-VAD-FMK, thus confirming the induction of apoptotic cell death by Bor (Fig. 2).

### Effects of Bortezomib on regulators and markers of apoptosis

**Fig. 3:**
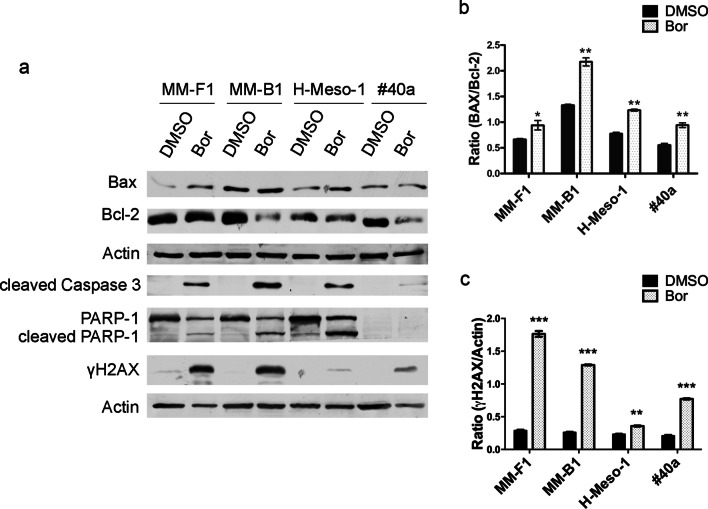
Effects of Bor on regulators and markers of apoptosis. **a** Western blotting analysis performed on human and mouse MM cells treated with 25 nM Bor or DMSO for 48 h. Actin was used as loading control. **b**, **c** Densitometric ratios between Bax and Bcl-2 (**b**), and between γH2AX and Actin (**c**). The ratios were calculated following densitometric analysis of the bands from two independent experiments and expressed as mean ± SD values (*p ≤ 0.05; **p ≤ 0.01; ***p ≤ 0.001)

To validate the effect of Bor on the induction of MM cells apoptosis, Bax, Bcl-2, cleaved-caspase 3, cleaved poly (ADP-ribose) polymerase-1 (PARP-1), and γH2AX levels were analyzed by Western blotting (Fig. 3). Levels of the pro-apoptotic Bax protein were increased by Bor in all cell lines and a concurrent decrease of the anti-apoptotic Bcl-2 protein was observed in Bor-treated MM-B1, H-Meso-1 and #40a cells (Fig. 3a). Accordingly, Bor treatment caused a significant increase of the Bax/Bcl-2 ratio in MM-F1 (*p* < 0.05), MM-B1 (*p* < 0.01), H-Meso-1 (*p* < 0.01), and #40a (*p* < 0.05) cell lines compared to controls (Fig. 3b).

Caspase 3 was activated by Bor treatment in all cell lines, as shown by the appearance of a specific band corresponding to the cleaved protein in Western blotting (Fig. 3a). Cleavage-activated caspase 3 induces the proteolytic inactivation of PARP-1, which in turn results in the inhibition of DNA repair and prevents DNA-repair-induced survival [20, 21]. Consistent with the induction of PARP-1 cleavage, Bor treatment increased the levels of the 89 kDa PARP fragment in MM-F1, MM-B1, and H-Meso-1 cells (Fig. 3a). Finally, apoptosis-related DNA fragmentation is known to trigger the phosphorylation of histone H2AX on Ser^139^, this phosphorylated form of H2AX being referred to as γH2AX [22, 23]. A significant increase of γH2AX levels was observed in all Bor-treated cell lines compared to those treated with DMSO (Fig. 3a, c). Remarkably, among the different MM cell lines, the lowest increase in γH2AX levels was observed in H-Meso-1, which, based on the results of FACS analysis, was also the cell line with the lower percentage of cells with a hypodiploid DNA content. Collectively these findings corroborate the activation of the apoptotic process in Bor-treated MM cells. Further, in order to validate the effect of Bor on proteasome inhibition in MM cells, lysates from Bor-treated cells were subjected to Western blotting and then probed with an anti-ubiquitin antibody. Upon Bor treatment, increased levels of ubiquitinated proteins were detected in all MM cell lines (Additional file 1: Figure S1), consistent with Bor-induced inhibition of proteasome activity [24].

### Effects of Bortezomib on markers of autophagy and endoplasmic reticulum stress

**Fig. 4:**
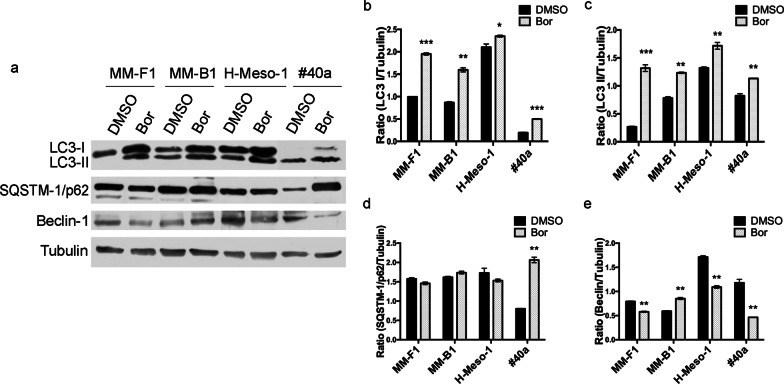
Effects of Bor on autophagy markers. **a** Western blotting analysis was performed on human and mouse MM cell lines treated with Bor 25 nM or DMSO for 48 h. Tubulin was used as a loading control. **b–e** Densitometric ratios between LC3-I and Tubulin (**b**), LC3-II and Tubulin (**c**), SQSTM-1/p62 and Tubulin (**d**), Beclin-1 and Tubulin (**e**). The ratios were calculated following densitometric analysis of the bands from two independent experiments and expressed as mean ± SD values (*p ≤ 0.05; **p ≤ 0.01; ***p ≤ 0.001)

It has been reported that Bor can modulate autophagy and that resistance to Bor treatment can be mediated by autophagy activation [25, 26]. To assess the effect of Bor on MM cell autophagy, the levels of the autophagosome marker Microtubule-Associated Protein Light Chain 3-II (LC3-II), the selective autophagy substrate SQSTM-1/p62 and Beclin-1 were evaluated by Western blotting (Fig. 4).

Changes in LC3-II levels must be interpreted considering those of the autophagy substrate SQSTM-1/p62. Indeed, increased LC3-II levels can be due to either increased autophagosome formation or decreased autophagosome clearance. Accordingly, the concurrent changes in LC3-II and SQSTM-1/p62 levels are used to discriminate between autophagy induction and decreased autophagic flux [27]. In the mouse #40a cell line Bor treatment increased both LC3-II and SQSTM-1/p62 levels, consistent with an inhibitory effect on autophagy (Fig. 4a, c, d). In the three human MM cell lines Bor increased LC3-II levels but had no significant effects on those of SQSTM-1/p62 (Fig. 4a, c, d). Moreover, the levels of Beclin-1, which is involved in many steps in autophagy pathways and is also known to play an anti-apoptotic role [28], were decreased by Bor in two out of the three human MM cell lines investigated as well as in the mouse cell line (Fig. 4a, e).

Although the effects of Bor on the three autophagy markers appeared to be cell line-dependent, the reported findings taken together suggest that Bor may interfere with the autophagic activity of human MM cell lines and decrease autophagy in the mouse MM cell line.

Treatment of cells with proteasome inhibitors such as Bor results in the accumulation of misfolded proteins within the endoplasmic reticulum (ER), which in turn can induce ER stress and activate the Unfolded Protein Response (UPR) [29]. The UPR aims to restore normal ER functions and enable cells to survive in ER stress conditions. However, severe or prolonged UPR activation can lead to apoptosis [29].

To determine the effects of Bor on ER stress and the UPR in MM cell lines, levels of the ER-resident chaperone Glucose-Regulated Protein 78 (GRP78/BiP) and CCAAT-enhancer-binding protein homologous protein (CHOP) were analyzed by Western blotting [29]. GRP78/BiP is known as a master regulator of ER stress with anti-apoptotic properties, while CHOP plays a main role in ER stress-induced apoptosis [30, 31].

**Fig. 5:**
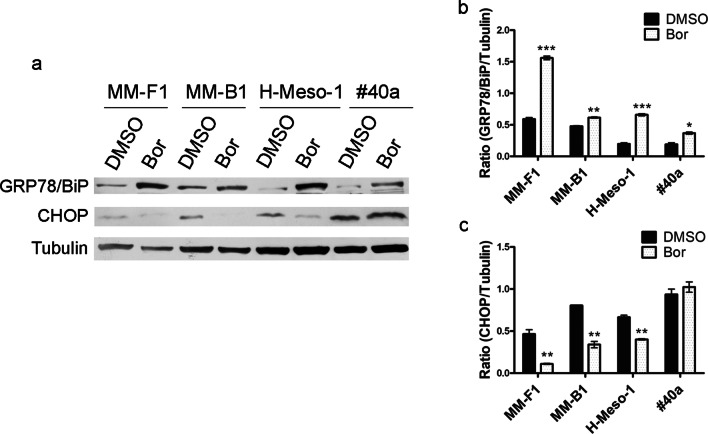
Effects of Bor on ER stress markers. **a** Western blotting analysis was performed on human and mouse MM cell lines treated with Bor 25 nM or DMSO for 48 h. Tubulin was used as a loading control. **b**, **c** Densitometric ratios between GRP78 and Tubulin (**b**), and between CHOP and Tubulin (**c**). The ratios were calculated following densitometric analysis of the bands from two independent experiments and expressed as mean ± SD values (*p ≤ 0.05; **p ≤ 0.01; ***p ≤ 0.001)

GRP78/BiP expression was significantly increased in all Bor-treated MM cell lines (Fig. 5a, b), consistent with the induction of ER stress and UPR activation [30]. On the other hand, Bor treatment decreased CHOP expression levels in all three human MM cell lines, and had no significant effect on CHOP expression in the mouse #40a cell line (Fig. 5a, c). The reported findings thus indicate that in Bor-treated MM cells activation of the UPR is not involved in the induction of apoptosis, but on the contrary, may participate to lower cells sensitivity to Bor cytotoxic effects.

### Effects of Bortezomib on ErbB receptors and activation of downstream pro-survival signaling effectors

**Fig. 6:**
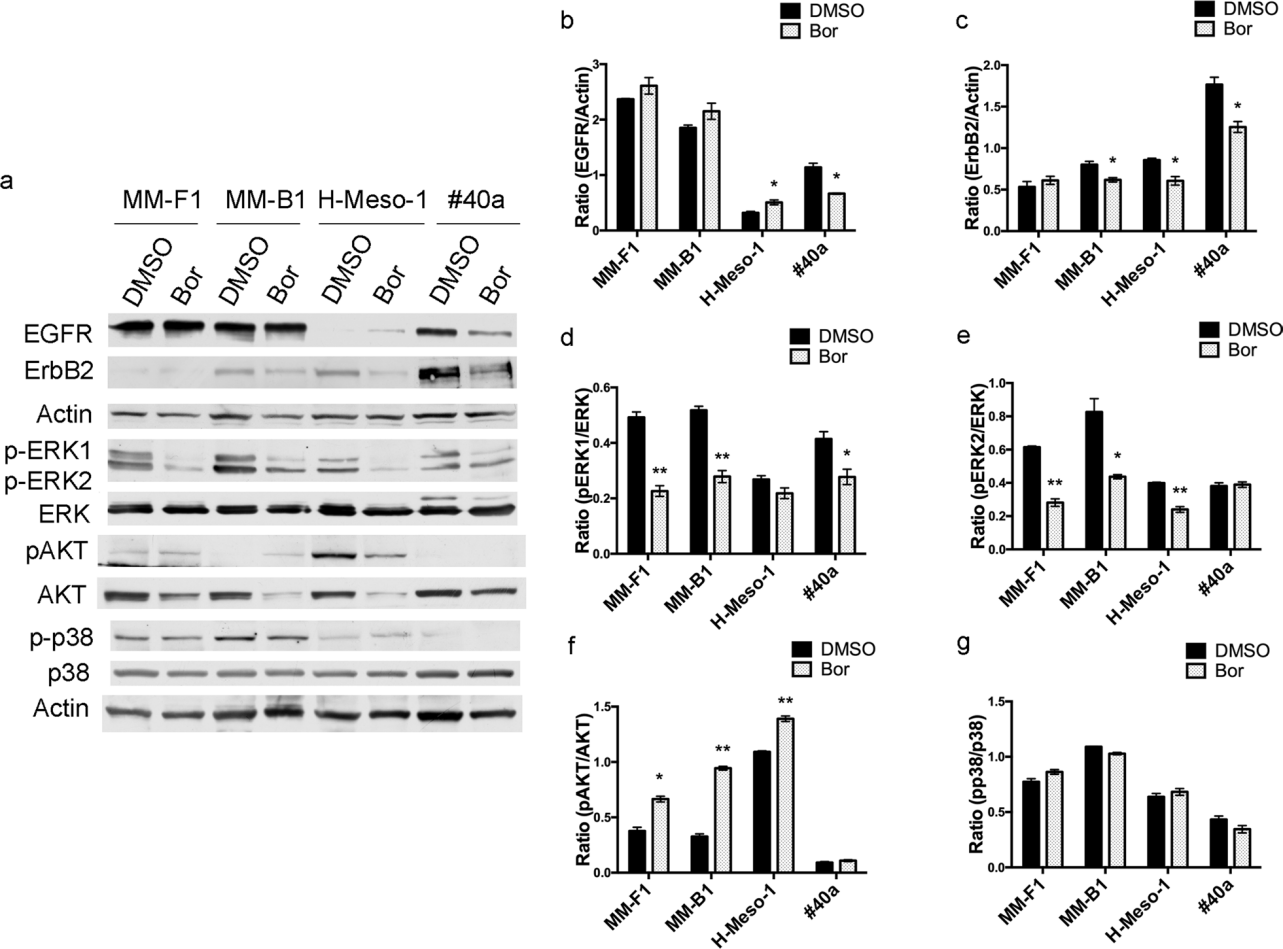
Effects of Bor on the expression EGFR and ErbB2 and activation of downstream signaling effectors. **a** Western blotting analysis was performed on human and mouse MM cell lines treated with 25 nM Bor or DMSO for 48 h. Actin was used as a loading control. **b–h** Densitometric ratios between EGFR and Actin (**b**), ErbB2 and Actin (**c**), pERK1 and ERK (**d**), pERK2 and ERK (**e**), pAKT and AKT (**f**), p-p38 and p38. **g** The ratios were calculated following densitometric analysis of the bands from two independent experiments and expressed as mean ± SD values (*p ≤ 0.05; **p ≤ 0.01; ***p ≤ 0.001)

Receptor tyrosine kinases of the ErbB family are overexpressed in MM. Their activation is linked to the MAP (Mitogen-Activated Protein) kinase and the phosphoinositide 3-kinase/AKT signal transduction cascades [32, 33]. Therefore, the expression of EGFR and ErbB2, as well as the expression and activation of the downstream pro-survival signaling effectors ERK1/2, AKT and p38 were analyzed by Western blotting (Fig. 6).

No significant differences in EGFR levels were observed between Bor- and DMSO-treated MM-F1 and MM-B1 cell lines, whereas Bor slightly increased EGFR expression in H-Meso-1 and diminished it in the #40a cell line (Fig. 6a, b). ErbB2 expression was decreased by Bor in MM-B1, H-Meso-1 and #40a cell lines, while no changes in ErbB2 levels were induced in MM-F1 cells (Fig. 6a, c). Therefore, Bor decreased the expression of at least one among EGFR and ErbB2 receptors in all cell lines except MM-F1.

Bor also reduced the activation of ERK1 and/or ERK2 in all cell lines. Indeed, upon Bor treatment, a decrease in the phosphorylated levels of both ERK1 and ERK2 was observed in MM-F1 and MM-B1 cell lines, and a decrease in the phosphorylation of one among the two kinases was detected in H-Meso-1 and #40a cell lines (Fig. 6a, d, e). On the other hand, AKT phosphorylation was increased by Bor in all treated human MM cell lines (Fig. 6a, f), and no significant effects of Bor were observed as regards p38 activation (Fig. 6a, g).

### Effect of Bortezomib on tumor growth in C57BL/6 mice intraperitoneally transplanted with syngeneic #40a MM cells

C57BL/6 mice (10 mice per group) were inoculated intraperitoneally with 1.5 × 10^6^ #40a cells and simultaneously treated intraperitoneally (i.p.) with 0.5 mg/kg of Bor dissolved in PBS-DMSO or with this vehicle alone as control (CTR). Treatments were then repeated twice a week. The growth of #40a cells in the peritoneum induces ascites. Accordingly, tumor growth was assessed by measuring the mice’s abdominal circumference before cell inoculation and weekly thereafter.

**Fig. 7:**
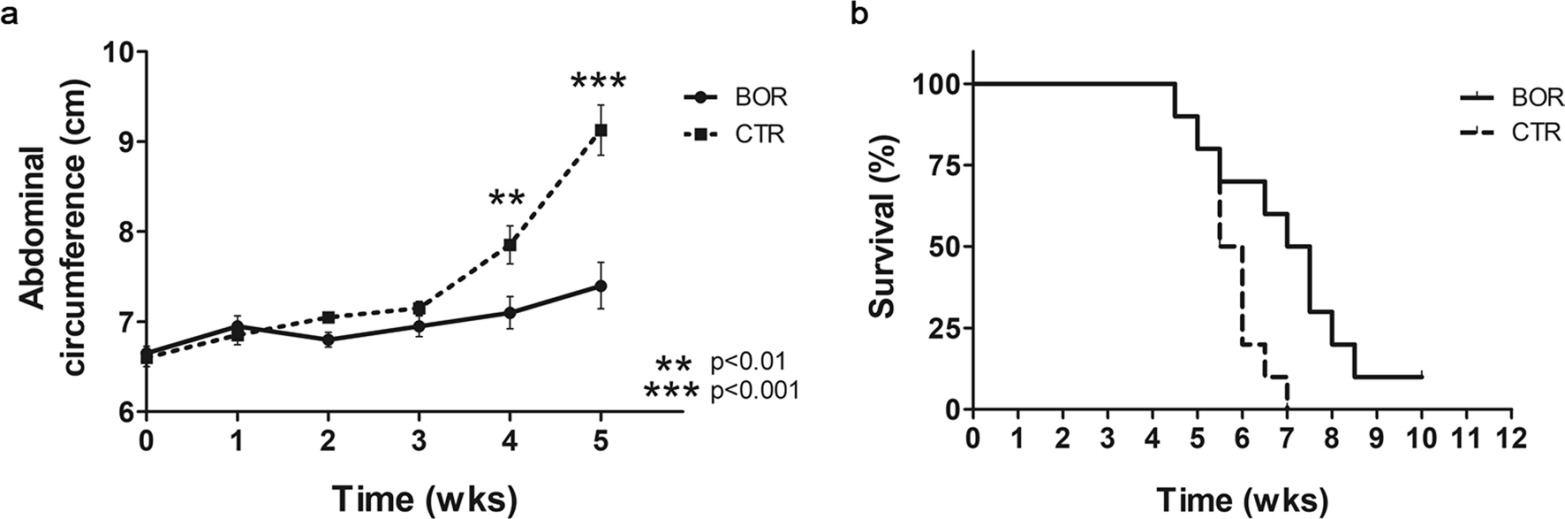
Bor reduced tumor growth and increased survival of C57BL/6 mice i.p. transplanted with syngeneic MM #40a cells. **a** Differences in mean abdominal circumferences between mice treated with Bor and control mice (CTR). **b** Differences in survival time of mice treated with Bor or with PBS-DMSO (CTR)

After four weeks of treatment, mice treated with Bor showed a significant decrease in the abdominal circumference compared to control mice (mean value 7.1 ± 0.6 vs. 7.9 ± 0.7 cm, *p* = 0.015) (Fig. 7a). After five weeks the mice’s abdominal circumference was further decreased in the Bor-treated vs. the CTR group (mean value 7.4 ± 0.8 vs. 9.1 ± 0.8 cm, *p* = 0.0003). After seven weeks from tumor cell transplantation, all CTR mice had been euthanized for the excessive size (≥ 12 cm) of the abdominal circumference, while 50% of mice in the Bor-treated group were still viable (Fig. 7b). Accordingly, the median survival of Bor-treated mice was significantly increased as compared to that of vehicle-treated mice (7.2 vs. 5.7 weeks; *p* = 0.02) (Fig. 7b). Based on the analysis of mice survival by the log-rank test (Mantel–Cox)*,* the Hazard Ratio for CTR mice vs. Bor-treated mice was 4.1 (Table 2).

**Table 2:** Analysis of mice survival after treatment with Bor or vehicle (CTR) by the log-rank test (Mantel–Cox)

Variable	Contrast	Hazard ratio	95% hazard ratio confidence limits		*p* value	Median survival (weeks)
			Lower	Upper		
Treatment	CTR vs. Bor	4.1	1.28	13.11	0.0172	5.75 vs. 7.25

### Effects of Bor on the frequency of immune cells recruited to the tumor microenvironment in C57BL/6 mice transplanted with syngeneic #40a MM cells

To evaluate the effect of Bor on phenotype and frequency of immune cells recruited to the tumor microenvironment, two groups of 5 mice were i.p. injected with #40a MM cells, treated with Bor or with PBS-DMSO as above, and then sacrificed after 30 days. The immune cells recruited in the ascitic fluids were collected by peritoneal lavage and analyzed by flow cytometry in comparison to spleen cells collected from the same mice, used as control. The gating strategy used to identify leukocyte subpopulations is illustrated in Additional file 1: Figure S2.

**Fig. 8:**
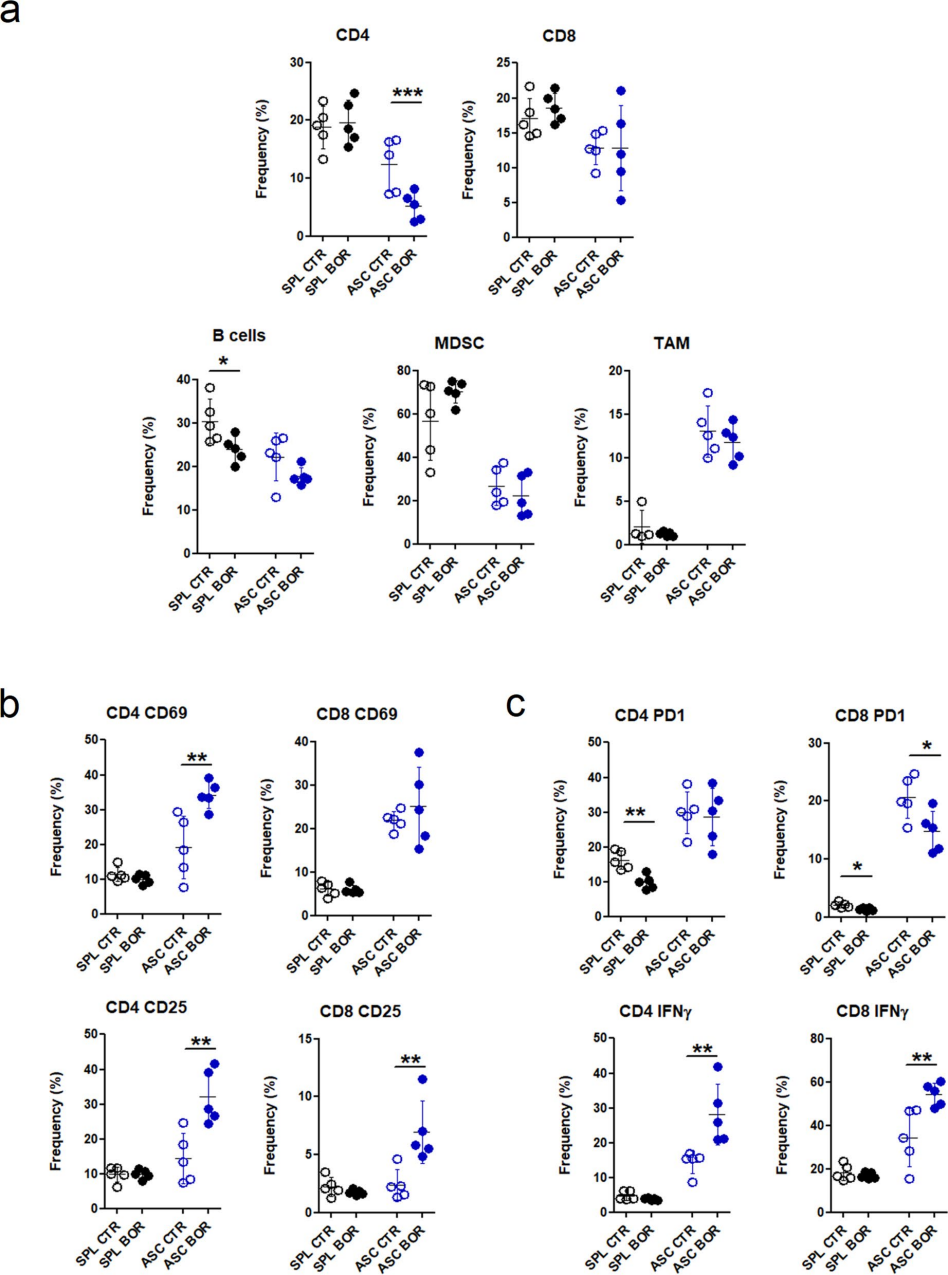
Effects of Bor on the frequency of immune cells and on the functional status of T cells recruited to the tumor microenvironment. Reported are mean ± SD values. Statistical significance of the differences observed between Bor-and vehicle-treated mice was evaluated by Student t-test (*p ≤ 0.05; **p ≤ 0.01; ***p ≤ 0.001). **a** Frequency of CD4^+^ and CD8^+^ T lymphocytes, B lymphocytes, Myeloid-derived suppressor cells (MDSCs) and Tumor associated macrophages (TAMs) in the spleen (SPL) and peritoneal ascitic fluid (ASC) of tumor-bearing mice (day 30) treated with Bor (n = 5) or vehicle (CTR) (n = 5), as assessed by flow cytometry. **b** Frequency of CD4^+^/CD69^+^, CD8^+^/CD69^+^ and of CD4^+^/CD25^+^, CD8^+^/CD25^+^ T lymphocytes in the spleen (SPL) or in the peritoneal ascitic fluid (ASC) of tumor-bearing mice (day 30) treated with Bor (n = 5) or with vehicle (CTR) (n = 5), as assessed by flow cytometry. **c** Frequency of CD4^+^/PD-1^+^, CD8^+^/PD-1^+^ and of CD4^+^/IFN-γ^+^, CD8^+^/IFN-γ^+^ T lymphocytes in the spleen (SPL) or in the peritoneal ascitic fluid (ASC) of tumor-bearing mice (day 30) treated with Bor (n = 5) or with vehicle (CTR) (n = 5), as assessed by flow cytometry

The frequency of CD4^+^ and CD8^+^ T lymphocytes collected from the spleen was similar in Bor-treated and vehicle-treated mice (Table 3). As for the lymphocytes infiltrating the ascitic fluid, the frequency of CD4^+^ T lymphocytes was decreased in Bor-treated vs. CTR mice (Bor 5.1 ± 2.4 vs. CTR 13.8 ± 2.7, *p* < 0.001), while similar frequencies were found for CD8^+^ T lymphocytes (Table 3 and Fig. 8a). The frequency of B lymphocytes, identified in flow cytometry as CD3^−^/B220^+^ cells, decreased significantly in the spleen of the Bor-treated group (Bor 23.9 ± 3.0 vs. CTR 30.4 ± 5.0, *p* < 0.05) and, although not statistically significant, a trend towards a decrease was also detected in Bor-treated mice ascitic fluids (Table 3 and Fig. 8a).

**Table 3:** Frequency of immune cells in spleen and peritoneal ascitic fluid of mice transplanted with syngeneic #40a MM cells

Cells	Spleen			Ascites		
	Control	Bor	*p* value	Control	Bor	*p* value
CD4^+^	18.8 ± 3.7	19.6 ± 3.8	ns	13.8 ± 2.7	5.1 ± 2.4	*p* ≤ 0.001
CD8^+^	18.0 ± 3.2	18.6 ± 2.1	ns	14.4 ± 1.1	12.8 ± 6.1	ns
B	30.4 ± 5.0	23.9 ± 3.0	*p* ≤ 0.05	22.2 ± 5.5	17.7 ± 2.1	ns
MDSC	56.6 ± 18.0	70.2 ± 5.2	ns	26.6 ± 8.8	22.1 ± 9.6	ns
TAM	3.6 ± 3.7	1.2 ± 0.3	ns	13.0 ± 2.9	11.8 ± 2.1	ns

*B* B cells, *MDSC* myeloid-derived suppressor cells, *TAM* tumor-associated macrophages, *ns* not significant

Myeloid-derived suppressor cells (MDSCs) constitute a population of cells involved in the negative regulation of the immune system, characterized by the ability to suppress T cell responses and to regulate innate immunity by modulating cytokine production by macrophages [34, 35]. The frequency of MDSCs, identified as CD3^+^/CD11b^HIGH^/F4/80^−^/Gr1^+^, was comparable in the spleen of vehicle- and Bor-treated mice; the frequency of MDSCs in ascitic fluids was lower than in spleens, but similar in the Bor- and CTR group (Table 3 and Fig. 8a). The frequency of tumor-associated macrophages (TAMs), identified as F4/80^+^/CD11b^+^, was instead higher in ascitic fluids than in spleens, but again similar among CTR and drug-treated mice (Table 3 and Fig. 8a).

### Effects of Bor on the functional status of T cells recruited to the tumor microenvironment in C57BL/6 mice transplanted with syngeneic #40a MM cells

The functional status of CD4^+^ and CD8^+^ T lymphocytes infiltrating the ascitic fluids of mice transplanted with #40a MM cells was investigated by flow cytometry in terms of activation status, production of cytokines, and expression of the immune checkpoint inhibitor Programmed Cell Death 1 (PD-1) protein.

Expression of the CD69 and CD25 T lymphocyte activation markers [36, 37] was first investigated. On the whole, comparable frequencies of T lymphocytes expressing CD25 and the early activation marker CD69 were found in spleens from Bor-treated and CTR mice (Table 4 and Fig. 8b). On the other hand, in Bor-treated mice the frequency of CD4^+^/CD69^+^ lymphocytes was more than doubled as compared to that of vehicle-treated mice (Bor 34.2 ± 3.9 vs. CTR 13.1 ± 9.0); no significant differences were instead observed in the frequency of ascitic CD8^+^/CD69^+^ cells among the two groups (Table 4 and Fig. 8b). As for the expression of the CD25 activation marker in ascitic fluid lymphocytes, Bor-treated mice showed a significant increase of both CD4^+^/CD25^+^ (Bor 32.1 ± 7.8 vs. CTR 14.5 ± 7.2) and CD8^+^/CD25^+^ T cells (Bor 6.9 ± 2.7 vs. CTR 2.4 ± 1.3) (Table 4 and Fig. 8b).

**Table 4:** Frequency of T lymphocytes expressing CD69 and CD25 activation markers in spleen and peritoneal ascitic fluid of C57BL/6 mice transplanted with #40a MM cells

Cells	Spleen			Ascites		
	Control	Bor	*p* value	Control	Bor	*p* value
CD4^+^CD69^+^	11.4 ± 2.1	10.0 ± 1.3	ns	13.1 ± 9.0	34.2 ± 3.9	*p* ≤ 0.01
CD8^+^CD69^+^	6.1 ± 1.6	5.9 ± 1.0	ns	21.8 ± 2.2	25.1 ± 8.9	ns
CD4^+^CD25^+^	9.8 ± 2.2	9.8 ± 1.4	ns	14.5 ± 7.2	32.1 ± 7.8	*p* ≤ 0.01
CD8^+^CD25^+^	2.3 ± 0.8	1.7 ± 0.2	ns	2.4 ± 1.3	6.9 ± 2.7	*p* ≤ 0.01

*ns* not significant

Activation of the PD-1 inhibitory receptor on T cells is a major mechanism by which cancer cells suppress T cell-mediated antitumor immune responses [38, 39]. The percentage of both CD4^+^/PD-1^+^ and CD8^+^/PD-1^+^ cells was reduced in the spleen of Bor-treated as compared to CTR mice (CD4^+^/PD-1^+^: Bor 9.9 ± 2.1 vs. CTR 16.2 ± 2.7; CD8^+^/PD-1^+^: Bor 1.3 ± 0.3 vs. CTR 2.1 ± 0.5). Moreover, lower amounts of CD8^+^/PD-1^+^ cells were also detected in ascitic fluids of drug-treated as compared to CTR mice (Bor 14.8 ± 3.4 vs. CTR 20.6 ± 3.6) (Table 5 and Fig. 8c). Furthermore, the percentage of T cells producing IFN-γ was significantly increased in both the CD4^+^ and CD8^+^ cell fractions collected from the ascitic fluids of Bor-treated mice (CD4^+^/IFN-γ^+^: Bor 28.2 ± 8.7 vs. CTR 14.3 ± 3.3; CD8^+^/IFN-γ^+^: Bor 54.2 ± 5.3 vs. CTR 34.4 ± 13.3) (Table 5 and Fig. 8c).

**Table 5:** Frequency of T lymphocytes expressing PD-1 and IFN-γ in spleen and peritoneal ascitic fluid of C57BL/6 mice transplanted with #40a MM cells

Cells	Spleen			Ascites		
	Control	Bor	*p* value	Control	Bor	*p* value
CD4^+^PD-1^+^	16.2 ± 2.7	9.9 ± 2.1	*p* ≤ 0.01	29.9 ± 6.0	28.6 ± 8.2	ns
CD4^+^IFN-γ^+^	4.8 ± 1.3	3.8 ± 0.4	ns	14.3 ± 3.3	28.2 ± 8.7	*p* ≤ 0.01
CD8^+^PD-1^+^	2.1 ± 0.5	1.3 ± 0.3	*p* ≤ 0.05	20.6 ± 3.6	14.8 ± 3.4	*p* ≤ 0.05
CD8^+^IFN-γ^+^	18.2 ± 3.6	16.9 ± 1.4	ns	34.4 ± 13.3	54.2 ± 5.3	*p* ≤ 0.01

*ns* not significant

According to the reported findings Bor treatment was able to sustain the activation of T cell-mediated immune responses in MM-bearing mice, associated with a reduction of the inhibitory marker PD-1 and an increased expression of the anticancer cytokine IFN-γ. These results, coupled with those obtained in in vitro studies, indicate that Bor can delay MM progression by exerting a direct growth inhibitory and pro-apoptotic effect on tumor cells as well as by promoting the activation of T cell-mediated immune responses.

## Discussion

Based on the in vitro results obtained using all four MM cell lines, Bor inhibited MM cell growth with IC_50_ values in the low-mid nanomolar range (10 < IC_50_ < 100 nM after 48 h and 10 < IC_50_ < 25 nM after 72 h of treatment). Such values are comparable with those reported in previous studies performed on MM cells [40, 41] and lower than the peak plasma levels obtained following i.v. Bor administration in patients with solid tumors [42]. According to trypan blue staining, MM cell growth inhibition was paralleled by increased cell death which, as evaluated based on the percentage of cells with a hypodiploid, sub-G1 DNA content, mainly occurred via apoptosis. Consistent with the induction of apoptotic cell death, Bor treatment increased the Bax/Bcl-2 ratio, as well as cleaved caspase 3 and γH2AX levels in all MM cell lines investigated [43].

Different mechanisms can concur to regulate the threshold for cell death upon Bor treatment. Previous studies have identified a high expression and activity of the proteasome [44, 45] and the dysregulation of the NOXA-dependent mitochondrial apoptotic pathway [46] as factors able to reduce the cytotoxic efficacy of Bor in MM cells. In this context, we here focused on two cellular processes that can be activated because of proteasomal inhibition: autophagy and the ER stress-triggered UPR.

Conflicting findings are reported as regards the effect of Bor on autophagy in cancer cells. Indeed, in some studies Bor treatment has been found to induce autophagy as an alternative mechanism of protein degradation aimed at protecting cancer cells from the toxic effects caused by proteasome inhibition, whereas opposing findings have been obtained in other studies [24, 26, 47]. The autophagic response to Bor treatment has not been previously investigated in MM cells to our knowledge. Although the effects of Bor on the autophagy markers appeared to be cell line-dependent, the concurrent changes of LC3-II, SQSTM-1/p62 and beclin-1 levels we observed in Bor-treated cells indicate that Bor interferes with the autophagic activity of human MM cell lines and decreases autophagy in the mouse MM cell line. Therefore, autophagy induction does not appear to play a role as a rescue mechanism that may decrease Bor efficacy on MM. On the other hand, based on the analysis of ER stress and UPR markers, a different rescue pathway appears activated by Bor in MM cells via the increase of the anti-apoptotic GRP78/BiP protein and the decrease of the pro-apoptotic CHOP protein.

The ER-resident chaperone GRP78/BiP acts as a master regulator of ER stress due to its ability to bind and inhibit the upstream activators of the UPR pathway, PKR-like ER kinase (PERK), inositol-requiring enzyme 1 (IRE1) and activating transcription factor 6 (ATF6), in a manner that is dependent on the quantity of unfolded proteins in the ER lumen. In ER stress conditions, unfolded proteins accumulate and are bound by GRP78/BiP, which in turn dissociates from PERK, IRE1 and ATF6, leading to UPR activation. While the different UPR pathways are primarily aimed at restoring cellular homeostasis, in case of severe or unresolved ER stress some UPR signaling branches will commit cells to death by apoptosis [48–50]. The CHOP transcription factor, whose expression is induced via PERK/ATF4, is a main player of the pro-apoptotic branch of the UPR, able to downregulate the expression of Bcl-2 family members, to enhance reactive oxygen species production and to exacerbate proteotoxic stress [48, 49].

According to our results, Bor increased GRP78/BiP levels in all MM cell lines investigated, consistent with ER stress induction and UPR activation [30]; however, the concurrent reduction of CHOP observed in the three human cell lines and the unchanged CHOP levels observed in the murine cell line indicate that such Bor-induced UPR activation has a pro-survival effect and likely participates to lower MM cells sensitivity to Bor cytotoxicity [51]. That evasion from UPR-mediated apoptosis can be involved in mediating resistance to Bor cytotoxicity in MM cells has been previously reported in a study performed using a MM cell line adapted to grow in the presence of increasing Bor concentrations (up to 40 nM) over a period of more than six months [52]. Our data, obtained using four different MM cell lines, suggest that primary resistance to UPR-mediated apoptosis may as well be an intrinsic common feature of MM cells. Remarkably, these findings have therapeutic implications, since they highlight that the combination with modulators of the UPR, some of which are already clinically available [48, 53], may represent a possible strategy to overcome Bor resistance in MM. In this respect, it is also worthy of note that growing evidence points to ER stress signaling as a pharmacological target for MM [54].

Considering the involvement of aberrant ErbB signaling in MM [32, 55] and the evidence that ErbB receptors levels are regulated, among the other mechanisms, via proteasomal degradation [56, 57], we also investigated whether Bor treatment could affect the expression of EGFR and ErbB2 and the activation of downstream pro-survival signaling effectors. The treatment resulted in reduced levels of at least one among EGFR and ErbB2 receptors in all cell lines except MM-F1. We recently reported a similar downregulation of EGFR and ErbB2 levels in Bor-treated head and neck carcinoma cells [24]. However, in the present study, Bor appeared to modulate EGFR levels in a cell line-dependent manner in MM, whereas more consistent effects were observed regarding ErbB2, whose levels were lowered by the proteasomal inhibitor in three out of the four MM cell lines investigated. The reduction of ErbB2 levels following treatment with proteasome inhibitors has also recently been observed in breast cancer cell lines, where it has been ascribed to both transcriptional downregulation and the induction of lysosomal degradation mechanisms [58].

As for the activation of the pro-survival pathways mediated by ERK1/2, AKT, and p38, Bor treatment was found to reduce the phospho-activation of ERK1 and/or ERK2 in all cell lines, had no significant effects on p38 phosphorylation levels, but increased the phosphorylation of AKT in the three human MM cell lines. In fact, Bor has been reported to either activate or inhibit AKT in a cell type-dependent manner, and the downregulation of phospho-AKT is regarded as an important determinant of Bor-induced apoptosis in cancer cells [24, 59, 60]. Worthy of note, in addition to its activation in response to growth factor receptors stimulation, AKT can also be activated by GRP78/BiP in ER stress conditions [61]. Moreover, the downregulation of AKT activity appears to play a determinant role in the induction of CHOP expression during ER stress [62]. Taken together, these considerations suggest that the type of ER stress induced by Bor in human MM cells may lead to the activation of AKT via the upregulation of GRP78/BiP; in turn, AKT may concur to reduce CHOP levels and impair the pro-apoptotic branch of the UPR. This latter hypothesis is indirectly supported by the observation that following Bor treatment CHOP expression was reduced in the three human MM cell lines showing increased phospho-AKT levels. While further studies are required to define the actual mechanisms responsible for Bor-induced AKT activation in MM cells, the reported findings point to the use AKT inhibitors as a therapeutic strategy aimed at increasing the apoptotic response of MM cells to Bor [60, 63].

The efficacy of Bor in mediating MM growth inhibition in vivo has been previously demonstrated in a study performed on immunodeficient (nude *xid*) mice carrying i.p. xenografts of human (REN) MM cells [40]. In the present study, we used a different model established in immunocompetent C57BL/6 mice, in which the growth of i.p. transplanted #40a MM cells reproducibly leads to ascites formation [32, 64–66]. The results obtained using this model confirm the ability of Bor to suppress MM growth in vivo and extend mice survival. In particular, while vehicle-treated mice had a median survival of 5.7 weeks, that of Bor-treated mice was of 7.2 weeks, with a Hazard Ratio for control vs. Bor-treated mice equal to 4.1.

Thanks to this model, we also evaluated the frequency and phenotype of immune cell populations recruited to the tumor site in mice transplanted with #40a MM cells and treated with Bor or vehicle for 30 days. In fact, although MM has been traditionally regarded as a non-immunogenic tumor, further studies have clarified that it is infiltrated by a non-negligible number of immune cells that, however, are functionally impaired via multiple factors operating in its immunosuppressive microenvironment [67–70]. In particular, the infiltration of MM tissues by hypofunctional lymphocytes has been reported by several authors and lately, due to the great clinical interest in ICIs, many studies have been focused on the involvement of lymphocyte inhibitory receptors such as PD-1 in MM immune escape [70–72].

When comparing the frequencies of CD4^+^, CD8^+^, B lymphocytes, MDSCs and TAMs in mice spleens with those in the ascitic fluids, we found significantly different values for MDSCs and TAMs only. At the tumor site MDSCs had a reduced frequency, while TAMs had an increased frequency as compared to the spleen. Still, the frequencies of both immune types were similar in the Bor-treated and CTR groups.

When comparing the frequency of the immune populations in the spleens of Bor-treated and CTR mice, the only significant change was a reduction in the frequency of B lymphocytes in the Bor-treated group, which however was not paralleled by a decreased frequency at the tumor site. Indeed, when comparing the frequency of the immune populations in the ascitic fluids of Bor- and vehicle-treated mice, the only significant change was a reduction in the frequency of CD4^+^ lymphocytes in the Bor-treated group.

More widespread differences were instead observed as regards the functional status of T lymphocytes from Bor-treated as compared to CTR mice. First, the ascitic fluids of Bor-treated mice had an increased frequency of both CD4^+^ and CD8^+^ T lymphocytes expressing the activation marker CD25, and an increased frequency of CD4^+^ T lymphocytes expressing the early activation marker CD69. Remarkably, this effect of Bor appeared specific for CD4^+^ and CD8^+^ cells recruited at the tumor site, since the same T populations collected from mice spleens showed no significant changes in the proportion of cells expressing the two markers. Next, consistent with the previous finding, Bor treatment resulted in a significant increase in the proportion of tumor-recruited CD4^+^ and CD8^+^ T cells expressing IFN-γ, a main cytokine marker for the activity of tumor-infiltrating lymphocytes [70], while it had no significant effects on the amount of IFN-γ^+^ T cells in the spleen. Finally, Bor treatment resulted in negative regulation of PD-1 expression in T cells. In contrast with the previous findings, the effect of Bor on the expression of the PD-1 immune checkpoint receptor was not limited to the lymphocytes recruited to the tumor site, since the proportion of CD8^+^/PD-1^+^ cells was reduced both in the spleen and ascitic fluid, and that of CD4^+^/PD-1^+^ cells in the spleen of the drug-treated mice. Overall, these findings indicate that Bor can sustain the activation of CD4^+^ and CD8^+^ T cells. Thus, besides its direct growth inhibitory and pro-apoptotic effects on tumor cells, Bor could delay MM progression by promoting the activation of T cell-mediated immune responses. Furthermore, by overcoming the exhaustion of T cells recruited in the tumor microenvironment, Bor could improve the outcomes of immunotherapy approaches in MM patients, as reported in preclinical studies performed on different cancer types [73, 74].

According to two phase II clinical trials where the therapeutic efficacy of Bor on MM was investigated alone (NCT00513877) or in combination with cisplatin (NCT00458913), the proteasomal inhibitor failed to significantly improve clinical outcomes [75, 76]. However, in both studies, Bor was administered i.v., whereas the therapeutic potential of intracavitary-administered Bor has not yet been investigated in patients with MM to our knowledge. Nonetheless, clinical results obtained in patients with recurrent ovarian cancer and myelomatous pleural effusions indicate that the intrapleural/intraperitoneal administration of Bor is feasible and has promising antitumor activity [77–79]. In fact, that the anticancer properties of Bor are currently underexploited for the treatment of solid tumors is evidenced by the many efforts which have recently been devoted to the development of Bor-based drug delivery systems that may improve its accumulation at the targeted tumor site [80]. Furthermore, a definitive assessment of Bor clinical utility for the treatment of MM would require the definition of criteria for the selection of patients to enroll in clinical trials [75]. An interesting avenue to explore in this regard may be to evaluate the possible impact of the mutational status of the de-ubiquitinating enzyme BRCA-1-associated protein BAP1 on the therapeutic outcome of Bor treatment. Indeed, BAP1 is a tumor suppressor frequently inactivated in several cancers including MM and its depletion has been reported to decrease tumor cells sensitivity to Bor, raising the hypothesis that this proteasomal inhibitor may have an increased efficacy in MM patients whose tumors express the wild type BAP1 protein [81, 82].

## Conclusions

The results of the present study support the use of Bor in MM and warrant further studies aimed at investigating the full potential of this drug through different delivery strategies and combination regimens. As regards the latter, our findings suggest that the efficacy of Bor on MM could be improved by the combination with agents such as UPR modulators and AKT-targeting agents and that, on the other hand, Bor could improve the therapeutic outcomes of immunotherapy and immune checkpoint inhibitors-based treatments.

## Materials and methods

### Antibodies and reagents

DMSO and Sulforhodamine B (SRB) were purchased from Sigma Aldrich (Milan, Italy). Bortezomib (Bor) was obtained from Selleck Chemical (Munich, Germany). Z-VAD-FMK was purchased by Calbiochem (San Diego, CA, USA). Antibodies against γH2AX, ERK, phospho-ERK, p38a/SAPK2a, and p38 MAPK (pT180/pY182) were obtained from BD Pharmingen (BD Biosciences, San Jose, CA, USA). Antibody against activated caspase 3 was obtained from Cell Signaling Technology (Danvers, MA, USA). Antibodies against Bax, Bcl-2, PARP-1 (F-2), AKT and phospho-AKT were obtained from Santa Cruz Biotechnology (Santa Cruz, CA, USA). Anti-ErbB2 (1:1000) and anti-EGFR (1:1000) antisera were provided by Dr. M. H. Kraus (University of Alabama, Birmingham, AL, USA) [83]. Antibodies against Beclin-1 and SQSTM-1/p62 were obtained from Abcam (Cambridge, United Kingdom) and the anti-LC3 antibody was purchased from Novus Biologicals (Littleton, CO, USA). Antibodies against ubiquitin, GRP78/BiP, and CHOP (GADD153) were obtained from ProteinTech Group (Rosemont, IL, USA). Antibody against tubulin was purchased from Immunological Sciences (Rome, Italy). Rabbit polyclonal anti-actin, the goat anti-mouse or -rabbit IgG peroxidase-conjugated secondary antibodies were obtained from Sigma-Aldrich.

Antibodies used in flow cytometry assays were produced by Sony Biotechnology and distributed by Cytosense (Milan, Italy): FITC anti-mouse CD3 (clone 17A2, Cat #1101020); PE anti-mouse F4/80 (clone BM8, Cat #1215550); PE/Cy7 anti-mouse CD25 (clone PC61, Cat #1110080); APC anti-mouse Ly-6G/Ly-6C (Gr-1) (clone RB6-8C5, Cat #1142060); Alexa Fluor 700 anti-mouse/human CD45R/B220 (clone RA3-6B2, Cat #1116160); Brilliant Violet 42 anti-mouse IFN-γ (clone XMG1.2, Cat #3129145); FITC anti-mouse CD4 (clone RM4-5, Cat #1102550); Alexa Fluor 647 anti-mouse CD279 (PD-1) (clone 29F.1A12, Cat #1276150); Brilliant Violet 785 anti-mouse CD69 (Cat #1122715); PE anti-mouse CD8a (clone 53-6.7, Cat #1103540); Brilliant Violet 510 anti-mouse/human CD11b (Cat #1106225).

### Cell lines and treatments

Human MM cell lines (H-Meso-1, MM-F1, MM-B1) were kindly provided by Prof. Antonio Procopio (Università Politecnica delle Marche, Ancona, Italy) and previously described [84, 85]. The murine MM cell line #40a was kindly provided by Dr. Agnes Kane (Department of Pathology and Laboratory Medicine, Brown University, Providence, RI, USA) and previously described by Goodglick et al. [86]. The #40a cell line was derived from the 40-cell line after two passages in the peritoneal cavity of syngeneic C57BL/6 mice following pristane administration one week before cells transplant. These passages allowed the selection of cells which reproducibly form ascites when intraperitoneally injected in mice [86]. #40a and H-Meso-1 cells have an epithelial morphology, while MM-F1 and MM-B1 cells have sarcomatous and biphasic features, respectively [86, 87]. Cells were maintained in DMEM (Dulbecco’s modified Eagle’s medium) containing 10% fetal bovine serum, 100 U/ml penicillin and 100 μg/ml streptomycin (complete medium). The cell lines were grown at 37 °C in a humidified incubator with an atmosphere of 5% CO_2_. Bor was dissolved in DMSO. For treatments, cells were incubated for the indicated times in the presence of Bor at various concentrations (dose range: 6.25–100 nM) or vehicle control (DMSO ≤ 0.1%).

### Sulforhodamine B (SRB) assay

Cells were seeded at 5 × 10^3^/well in 96-well plates and incubated at 37 °C to allow cell attachment. After 24 h, the medium was changed and cells were incubated for 24, 48 and 72 h with Bor (6.25–100 nM) or with DMSO. Cells were then fixed with cold trichloroacetic acid (final concentration 10%) for 1 h at 4 °C. The assay was then performed as previously described [24]. The percentage survival of the cultures treated with Bor was calculated by normalization of their O.D. values to those of the control cultures treated with DMSO [32]. The experiments were performed in triplicate and repeated three times.

### Trypan blue exclusion assay

Cells were seeded at 5 × 10^4^/well in 24-well plates and incubated at 37 °C to allow cells attachment. After 24 h, the medium was changed and cells were incubated for 24, 48, and 72 h with Bor (6.25–100 nM) or DMSO. Adherent as well as suspended cells of each well were then harvested, stained with trypan blue (Sigma-Aldrich, Milan, Italy) and counted under a light optical microscope [88]. The experiments were repeated three times, and the percentage of dead cells was calculated compared with the total cell number [64].

### FACS analysis of DNA content

Asynchronized log-phase growing cells (60% confluent, approximately 2.5 × 10^5^cells/well in 6-well plates) were treated with Bor or DMSO in a complete culture medium. ZVAD-FMK was used at a final concentration of 40 μM for 2 h before adding Bor. After 48 h, adherent and suspended cells were harvested, centrifuged at 1500 rpm for 10 min, and washed twice with cold phosphate-buffered saline (PBS). The cells were then marked with propidium iodide and analyzed by flow cytometry as previously described [89], using a FACS-Calibur cytometer running CellQuest Pro 5.2 software (BD Biosciences, San Jose, CA, USA).

### Western blotting

Approximately 1 × 10^6^ cells were seeded in 100 mm tissue culture dishes 24 h before the addition of 12.5 or 25 nM Bor or DMSO. After 14, 24 or 48 h of treatment, cells were harvested, washed twice with cold PBS, and lysed in RIPA buffer as previously described [90, 91]. For immunoblotting analysis, 80 μg of cell lysates were resolved in 10, 12 or 15% SDS-PAGE and then transferred to nitrocellulose membranes [92, 93]. Equal loading and transfer of proteins were verified by Ponceau red staining of the membranes. After blocking, the membranes were incubated overnight at 4 °C with specific primary antibodies at 1–2 µg/ml concentrations, then washed and incubated with goat anti-mouse or anti-rabbit IgG peroxidase-conjugated antibodies and finally developed by chemiluminescence as previously described [24]. Protein expression was detected using the enhanced chemiluminescence system ECL LiteAblot (Euroclone, Milan, Italy). Actin or Tubulin was used as control. The densitometric analysis of autoradiographic bands was performed with the ImageJ 1.53e software (National Institutes of Health, United States) after blot scanning.

### In vivo treatment of C57BL/6 mice intraperitoneally transplanted with #40a cells

Groups of 6-to-8-weeks-old C57BL/6 mice (10 mice for each group) were intraperitoneally (i.p.) inoculated with 0.2 ml of suspension containing 1.5 × 10^6^ #40a cells in phosphate-buffered saline (PBS) 1 week after pristane i.p. injection (500 µl). Mice were treated i.p. with Bor (0.5 mg/kg in PBS-DMSO), or PBS-DMSO (400 µl). The treatments were started simultaneously with the inoculation of MM cells and repeated twice a week thereafter.

The investigation was conducted in accordance with ethical standards and according to the Declaration of Helsinki. A veterinary surgeon was present during the experiments. The animal care, both before and after the experiments, was performed by trained personnel only. Mice were bred under pathogen-free conditions in the animal facilities of the University of Rome “Tor Vergata” and handled in compliance with European Union and institutional standards for animal research. The work was conducted with the formal approval of the local animal care committees (institutional and national), and animal experiments were registered as legislation requires (Authorization from Ministry of Health no. 179_2020-PR).

### Analysis of Bor antitumor activity in vivo

#40a cells growth in the peritoneum induces ascites. Accordingly, the abdominal circumference of mice was monitored before the inoculation of cells and then every week. Tumor-bearing mice were euthanized at the first signs of distress or when their abdominal circumference reached 12 cm.

### Phenotypical analysis of immune cells from the ascitic fluid of C57BL/6 mice transplanted with #40a cells

Groups of 6-to-8-weeks-old C57BL/6 mice (5 mice for each group) were i.p. inoculated with #40a cells and treated with Bor or PBS-DMSO as described above. The treatments were started simultaneously with the inoculation of MM cells and lasted up to 30 days, when mice were euthanized. To collect tumor cells and immune cells recruited to the tumor microenvironment, a peritoneal lavage was performed by injecting 5 ml of cold PBS into the peritoneum and gently massaging the peritoneum for 30 s to dislodge the tumor and immune cells into the PBS that was then recovered. These cell suspensions were then processed by density gradient centrifugation with Pancoll separating solution (PAN-Biotech Cat #P04-69600) to separate mononuclear immune cells from tumor cells and erythrocytes present in the ascitic fluid. Spleens were also collected and processed onto a cell strainer to obtain a cell suspension. Red blood cells lysis was performed to eliminate contaminating erythrocytes (Roche Cat #11814389001).

Multicolor flow cytometry was then performed to define the phenotype of immune cells contained in the ascitic fluid as compared to spleen cells. Dead cells were excluded using fixable Viability Dye eFluor780 (eBioscience, San Diego, California, Cat #65-0865-14) for 30 min at room temperature. Surface staining was performed by incubating the cells with antibodies for 20 min at 4 °C in PBS containing FBS 2%. Intracellular staining was achieved using True-Nuclear™ Transcription Factor Buffer Set according to the manufacturer's instructions (Sony Biotechnology, Cat #2722005). Before IFN-γ staining, cells were stimulated for 4 h at 37 °C with Cell Stimulation Cocktail plus protein transport inhibitors (eBioscience Cat #00-4975-03). Data were acquired and analyzed with CytExpert Acquisition and Analysis Software Version 2.5 onto a Cytoflex instrument (Beckman Coulter). For the gating strategy, lymphocytes were selected based on SSC-A and FSC-A and on the exclusion of dead cells.

### Statistical analysis

Statistical analysis was performed using the GraphPad Prism software updated to version 5.0 (La Jolla, California). Data obtained in cell growth, cell death, and cell cycle studies were preliminarily verified using the Kolmogorov–Smirnov test, and the data sets were analyzed by one-way analysis of variance (ANOVA) followed by the Newman-Keuls test. Differences in the intensity of the immunoreactive bands were evaluated using two-tailed Student’s t-test. Survival curves and differences in mice abdominal circumferences were analyzed using the Kaplan–Meier method and compared with a log-rank test (Mantel–Cox) [94]. Differences in the frequency of stained cells in flow cytometric assays were evaluated using two-tailed Student’s t-test. In all studies, the significance threshold was set at *p* ≤ 0.05.

## Supplementary Information

**Additional file 1. Figure S1:** Effect of Bor on protein ubiquitination. **Figure S2:** Gating strategies for the identification of leukocyte subpopulations.

## Data Availability

All data generated or analyzed during this study are included in this published article [and its supplementary information files].
