# Supplementary material for: Antitumoral effects of Bortezomib in malignant mesothelioma: evidence of mild endoplasmic reticulum stress in vitro and activation of T cell response in vivo
Source: Biol Direct. 2023 Apr 17;18:17. doi: 10.1186/s13062-023-00374-w (PMC10111665; doi:10.1186/s13062-023-00374-w)
Supplement: Supplementary file 1 — Additional file 1. Figure S1: Effect of Bor on protein ubiquitination. Figure S2: Gating strategies for the identification of leukocyte subpopulations. [file 13062_2023_374_MOESM1_ESM.docx]

**Supplementary Information**

**Additional File 1**


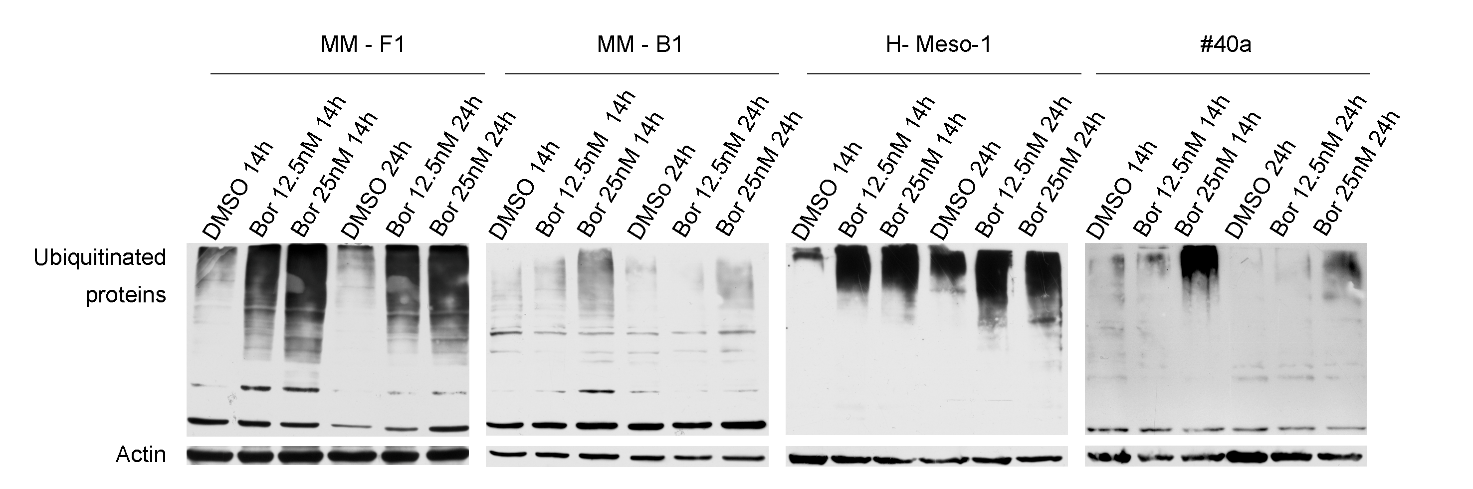


**Figure S1: Effect of Bor on protein ubiquitination.** Western blotting analysis was performed on lysates from MM cell lines treated with Bor (12.5 and 25 nM) or DMSO for 14 or 24 h. Blots were probed with an anti-ubiquitin antibody. Actin was used as loading control.


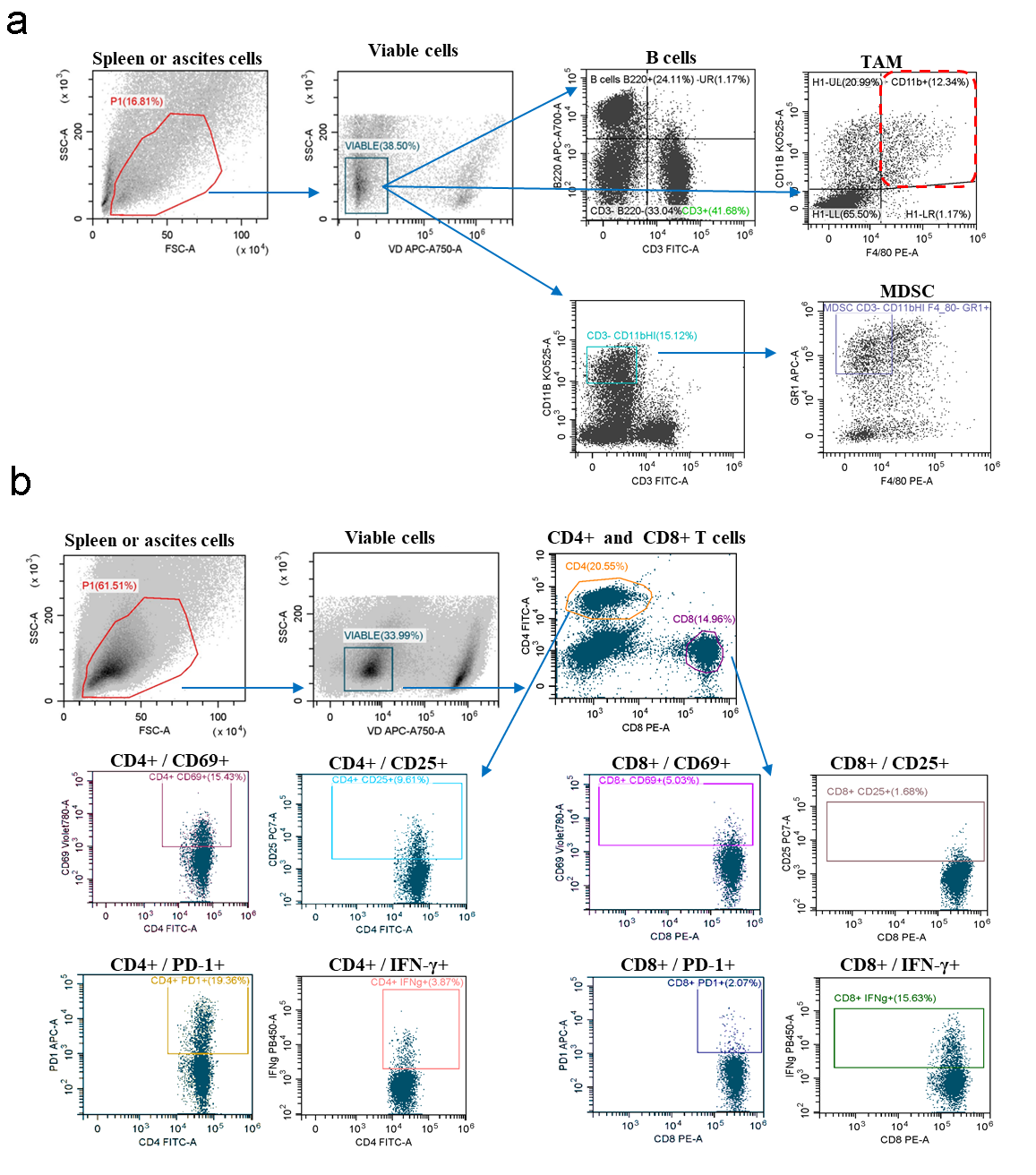


**Figure S2: Gating strategies for the identification of leukocyte subpopulations.** a) Flow cytometry gating strategy used to define B cells (CD3^-^B220^+^), MDSCs (CD3^-^CD11b^HI^F4/80^-^Gr-1^+^) and TAMs (F4/80^+^CD11b^+^) in the spleens or ascitic fluids of C57BL/6 mice transplanted with #40a MM cells. b) Flow cytometry gating strategy used to define CD4 and CD8 T cells (CD4+ or CD8+), activated T cells (CD4^+^CD69^+^ or CD4^+^CD25^+^or CD8^+^CD69^+^ or CD8^+^CD25^+^), and expression of PD-1 and IFN-γ on CD4 and CD8 T cells in the spleens or ascitic fluids of C57BL/6 mice transplanted with #40a MM cells. Leukcocytes were gated based on SSC-A versus FSC-A, and dead cells were excluded with Fixable Viability Dye.
